# Reverse stress testing interbank networks

**DOI:** 10.1038/s41598-017-14470-1

**Published:** 2017-11-15

**Authors:** Daniel Grigat, Fabio Caccioli

**Affiliations:** 1 University College London, Department of Computer Science, London, WC1E 6BT United Kingdom; 2 London School of Economics, Systemic Risk Centre, London, WC2A 2AE United Kingdom

## Abstract

We reverse engineer dynamics of financial contagion to find the scenario of smallest exogenous shock that, should it occur, would lead to a given final systemic loss. This reverse stress test can be used to identify the potential triggers of systemic events, and it removes the arbitrariness in the selection of shock scenarios in stress testing. We consider in particular the case of distress propagation in an interbank market, and we study a network of 44 European banks, which we reconstruct using data collected from banks statements. By looking at the distribution across banks of the size of smallest exogenous shocks we rank banks in terms of their systemic importance, and we show the effectiveness of a policy with capital requirements based on this ranking. We also study the properties of smallest exogenous shocks as a function of the parameters that determine the endogenous amplification of shocks. We find that the size of smallest exogenous shocks reduces and that the distribution across banks becomes more localized as the system becomes more unstable.

## Introduction

Systemic risk – the risk associated with the occurrence of a catastrophic breakdown of the financial system – arises endogenously from interactions between the participants that operate in financial markets. Because some types of interactions between financial institutions (in the following banks for brevity) can be modeled in terms of dynamical processes on networks, a growing body of literature has focused on the study of contagion and distress propagation in financial networks^1–3^. This research began in the year 2000 with the work of Allen and Gale, who showed that the topology of financial networks influences financial contagion^4^. Many different algorithms have since then been developed to model the propagation of distress between banks under different assumptions as well as to study the relation between the structure of a financial network and its stability (see for instance^3,5–21^). In this respect, significant progress has been made in the identification of the main drivers of financial contagion and in the design of new stress test frameworks that, at odds with standard micro-prudential tools, do account for interactions between banks^13,21–26^.

While the focus of research carried out so far has been mainly that of developing models to understand how exogenous shocks are amplified by the endogenous dynamics of the system, here we look at the reverse problem. We compute the time trajectories of smallest shocks that need to affect banks to produce a final loss of equity larger than a given threshold, which we therefore refer to as worst case shocks. The solution of this reverse problem is useful to identify stress scenarios whose occurrence would lead to systemic events, thus identifying the vulnerabilities of a financial system.

At the level of individual institutions, reverse stress testing is a regulatory requirement in the United Kingdom (UK) and the European Union. The Financial Services Authority, one of the UK’s financial regulators, describes it as a complementary exercise to general stress and scenario testing. In standard stress testing a forward-looking methodology is employed, in which scenarios are selected to predict their potential impact upon the financial health of banks. Reverse stress testing on the other hand looks backward by identifying the scenarios that cause a specific loss to a bank. This way of identifying stress scenarios is the major advantage of reverse stress testing. Instead of relying on the judgement of experts to select scenarios, the most dangerous scenarios are automatically identified.

Previous work in this area has focused on developing reverse stress testing frameworks that are intended to be used for the risk analyses of individual institutions rather than of the financial system as a whole^27^. Some studies are dedicated to optimizing scenario selection, and defining probability distributions of the numerous intertwined driving variables across asset classes. For two recent reviews see^3,28^ and for a mathematical approach to worst case scenario selection see^29^. However, we were not able to find any previous research on reverse stress testing in interbank networks that investigates systemic risk.

In this paper we present as a case study a reverse stress test analysis of a system composed of the 44 European banks that are the constituents of the STOXX Europe 600 Banks index. This index is the major equity benchmark of the most significant financial institutions in Europe. For each bank we collected data on total interbank lending, total interbank borrowing and Tier 1 equity capital. We used the RAS algorithm^30^ to reconstruct the matrix of interbank exposures. We then computed the worst case shocks under a linear model of distress propagation, the so-called DebtRank^13^. We chose this contagion algorithm because of its simplicity and because it can be considered a first-order approximation for a more generic class of contagion algorithms^31,32^.

It must be noted that the importance of interbank exposures for financial contagion has been questioned^33,34^. In spite of this there still are valid reasons to look at interbank exposures networks. First of all, they can amplify contagion due to other mechanism^35,36^. Second, the result about the limited effect of interbank exposure refers to the situation in which stress is propagated from a borrower to the lender only after the default of the borrower. However in practice, as pointed out in^34^, losses can occur even in absence of defaults because of credit quality devaluation – the situation in which lenders revalue their interbank assets because they perceive a decline in the ability of their counterparties to repay their debt – which DebtRank aims at capturing in a crude way^37^.

Our main results are the following:

- We show that as the largest eigenvalue of the matrix of interbank leverages increases the worst case shocks become smaller and concentrated in a smaller set of banks;
- We compute the distribution across banks of worst case shock sizes, thus providing a ranking of banks in terms of their systemic importance;
- We show that the obtained ranking can be used to make the system more robust through the implementation of targeted capital requirement policies.

It must be stressed that all our results are derived using a very specific mechanics of distress propagation, the so-called DebtRank, and as such they are affected by this choice. In particular, the linear assumption of DebtRank tends to overestimate losses with respect to other contagion mechanisms proposed in the literature^38^. This implies that the worst case shocks that we compute are the smallest across the different contagion algorithms and could be used to provide lower bounds for the size of the exogenous shocks needed to produce systemic events.

Beyond the specific results we obtain, we regard as the main contribution of the paper that of employing contagion algorithms to reverse engineer contagion dynamics in complex systems. This approach is inspired by (network) control theory, which is a methodology from engineering recently applied to complex systems^39^. In control theory the goal is to drive a system (in our case a network representing interbank lending between banks) from an initial state to a desired target state (in our case to a minimum level of financial losses) with the least effort (in our case exogenous shocks to the balance sheets of banks).

## Results

### Problem set-up

We consider a system of *N* banks that interact through a network of mutual exposures (interbank assets and liabilities). A bank is financially characterised through numerous positions on its balance sheet, the three most important positions for our study are the interbank assets and liabilities as well as the equity. The interbank assets correspond to the total sum of loans that a bank extended to other banks (its interbank counterparties), whereas interbank liabilities are the total sum of loans borrowed from other banks. The equity is the difference between assets and liabilities and describes the capital buffer that can be used to absorb losses. In the case of systemic risk one of the most common losses is that due to the default of a borrower, that is the inability of a bank to repay (part of) its loans.

In the following we consider a discrete time dynamic for the value of banks’ portfolios, and we denote by *A*_*ij*_(*t*) the value of the exposure of bank *i* to bank *j* at time *t*, by *E*_*i*_(*t*) the equity of bank *i* at time *t*, by ${A}_{i}^{{\rm{ext}}}(t)$ the value of external (i.e. non interbank) assets of bank *i* at time *t*, and finally by *L*_*i*_ the liabilities of bank *i*, that we assume to be constant over time. A further assumption is that banks do not rebalance their portfolio (i.e. the number of shares they own of an asset is assumed to be constant), so that the changes in the balance sheet of a bank are only due to changes in the price of the bank’s assets.

From the balance sheet identity we have that

1 $${E}_{i}(t)=\sum _{j}\,{A}_{ij}(t)+{A}_{i}^{{\rm{ext}}}(t)-{L}_{i}\mathrm{.}$$

We now consider a situation in which the value of external assets is subject to random market fluctuations, while the value of the interbank assets of a bank at time *t* depends on the equity of its counterparties at time *t* − 1. Following^13,21^ we assume that the relative devaluation of an interbank asset is proportional to the relative devaluation of the equity of the counterparty:

2 $$\frac{{A}_{ij}(t)-{A}_{ij}\mathrm{(0)}}{{A}_{ij}\mathrm{(0)}}=\beta \frac{{E}_{j}(t-\mathrm{1)}-{E}_{j}\mathrm{(0)}}{{E}_{j}\mathrm{(0)}},$$

where *β* ∈ [0, 1] is a positive constant related to the strength of contagion between counterparties. More specifically, *β* represents the percentage loss experienced by a lender when the corresponding borrower looses 1% of its equity. This also means that *β* is the fraction of interbank loan that is lost upon the default of the borrower, i.e. the loss given default. Therefore the equity of bank *i* evolves in discrete time according to

3 $${E}_{i}(t)=\beta \,\sum _{j}\,\frac{{A}_{ij}\mathrm{(0)}{E}_{j}(t-\mathrm{1)}}{{E}_{j}\mathrm{(0)}}+{A}_{i}^{{\rm{ext}}}(t)-{L}_{i}\mathrm{.}$$

Following^21^ we now define ${h}_{i}(t)=\frac{{E}_{i}\mathrm{(0)}-{E}_{i}(t)}{{E}_{i}\mathrm{(0)}}$ and ${{\rm{\Lambda }}}_{ij}=\frac{{A}_{ij}\mathrm{(0)}}{{E}_{i}\mathrm{(0)}}$, so that

4 $${h}_{i}(t)=\beta \,\sum _{j}\,{{\rm{\Lambda }}}_{ij}{h}_{j}(t-\mathrm{1)}+\frac{{A}_{i}^{{\rm{ext}}}\mathrm{(0)}-{A}_{i}^{{\rm{ext}}}(t)}{{E}_{i}\mathrm{(0)}}\mathrm{.}$$

The quantity Λ_*ij*_ represents the importance for bank *i* of its interbank asset associated with bank *j*, as measured in terms of *i*’s equity. In particular, if the value of the interbank asset drops by 1%, bank *i* would experience a loss of Λ_*ij*_% of its equity. For this reason, Λ_*ij*_ is referred to as the matrix of interbank leverages^21^.

In this paper we focus on direct exposures as a means of financial contagion. However, we note that our framework could be extended to other cases, like those of rollover risk^40^ or overlapping portfolios^24^. In the case of rollover risk, internal assets would be short term interbank assets, and the matrix of interbank leverage would be replaced by its transpose. In the case of overlapping portfolios, internal assets would instead be assets that can be liquidated by banks during a fire sale, external assets would be very illiquid assets that are not marketable, and the matrix of interbank leverage would be replaced by the matrix of overlaps between banks portfolios.

We now further define ${u}_{i}(t)=\frac{{A}_{i}^{{\rm{ext}}}\mathrm{(0)}-{A}_{i}^{{\rm{ext}}}(t)}{{E}_{i}\mathrm{(0)}}$, which represents the contribution to the relative equity loss of bank *i* due to shocks to its external assets between times 0 and *t*, so that

5 $${h}_{i}(t)=\beta \,\sum _{j}\,{{\rm{\Lambda }}}_{ij}{h}_{j}(t-\mathrm{1)}+{u}_{i}(t\mathrm{).}$$

Similarly to^41^ we can thus measure the impact of shocks on the capital of each bank and compute their propagation across time. We now imagine a situation in which we want to reverse stress test the system over a time horizon *T*. In particular, we assume to be at time *t* = 0 and we look for trajectories of shocks $\{\overrightarrow{u}\mathrm{(1)},\overrightarrow{u}\mathrm{(2)},\ldots ,\overrightarrow{u}(T)\}$ to external assets that can lead at time *T* to losses equal or greater than a given threshold, i.e. such that

6 $${h}_{i}(T)=\sum _{t=1}^{T}\,\sum _{j=1}^{N}\,{\beta }^{T-t}\,{({{\rm{\Lambda }}}^{T-t})}_{ij}\,{u}_{j}(t)\ge {\ell }_{i},$$

with *i* ∈ {1, 2, …, *n*}, and where we have denoted by ${\ell }_{i}$ the threshold associated with the loss of bank *i*. We also note that ${{\rm{\Lambda }}}_{ij}^{0}={\delta }_{ij}$, the Kronecker delta; in other words Λ^0^ is the identity matrix. There are clearly many possible trajectories that satisfy the constraints (6); here we are interested in identifying those that minimize fluctuations of relative losses on external assets over time, i.e. for which the following quantity is minimized:

7 $$K\equiv \sum _{i=1}^{N}\,\sum _{t=1}^{T}\,{({u}_{i}(t)-{u}_{i}(t-\mathrm{1)})}^{2}$$

The cost function *K* can be interpreted as the aggregate size of the exogenous shock affecting the system. We select this cost function because we seek to identity the smallest exogenous shocks that should hit the system to cause a given final loss. We refer to these shocks as “worst case shocks”. Given that here we do not make a distinction between positive or negative shocks, minimizing the cost function *K* ensures that we can identify the trajectories of exogenous shocks that correspond to the minimal deviation from the status quo.

In the following we will also refer to the contribution of a bank *i* to the aggregate shock as “nodal shock”, that is the total shock affecting bank *i*:

8 $${K}_{i}\equiv \sum _{t=1}^{T}\,{({u}_{i}(t)-{u}_{i}(t-\mathrm{1)})}^{2}\mathrm{.}$$

In summary, we are interested in solving the following optimization problem

9 $$\begin{array}{l}\,\,{\rm{\min }}\,(\frac{1}{2}\,\sum _{i=1}^{N}\,\sum _{t=1}^{T}\,{\rm{\Delta }}{u}_{i}{(t)}^{2}),\\ {\rm{s}}.{\rm{t}}.\,\sum _{t=1}^{T}\,{\beta }^{T-t}\,{({{\rm{\Lambda }}}^{T-t})}_{ij}\,{u}_{j}(t)\ge {\ell }_{i},\,\forall i\mathrm{.}\end{array}$$

where we have defined Δ*u*_*i*_(*t*) = *u*_*i*_(*t*) − *u*_*i*_(*t* − 1) and assumed *u*_*i*_(0) = 0 for all *i*. Δ*u*_*i*_(*t*) represents the loss due to shocks on external assets experienced by bank *i* between times *t* − 1 and *t*. The optimization problem can be more conveniently written in terms of the only variables Δ*u*’s as

10 $$\begin{array}{l}\quad \quad {\rm{\min }}\,(\frac{1}{2}\,\sum _{i=1}^{N}\,\sum _{t=1}^{T}\,{\rm{\Delta }}{u}_{i}{(t)}^{2}),\\ {\rm{s}}\mathrm{.}{\rm{t}}\mathrm{.}\,\sum _{t=1}^{T}\,{\beta }^{T-t}\,\sum _{s=1}^{t}\,{({{\rm{\Lambda }}}^{T-t})}_{ij}\,{\rm{\Delta }}{u}_{j}(s)\ge {\ell }_{i},\,\forall i\mathrm{.}\end{array}$$

### Homogeneous system

In order to develop an intuition on the behavior of the solutions of (10), we first consider the simple case of a homogeneous system in which all banks have the same interbank leverage *c*, i.e. we assume that the matrix Λ is such that ${\sum }_{j}\,{{\rm{\Lambda }}}_{ij}=c$ for all *i*. In this case all banks are the same, they are subject to the same dynamic and to the same constraint. Therefore, by symmetry, also the trajectories of worst case shocks are the same for all banks. The optimization problem (10) reduces then to

11 $$\begin{array}{l}{\rm{\min }}\,(\frac{1}{2}\,\sum _{t=1}^{T}\,{\rm{\Delta }}u{(t)}^{2}),\\ {\rm{s}}\mathrm{.}{\rm{t}}\mathrm{.}\,\sum _{s=1}^{T}\,\sum _{t=s}^{T}\,{\lambda }^{T-t}{\rm{\Delta }}u(s)\ge \ell ,\end{array}$$

where we have defined *λ* = *βc* and Δ*u*(*t*) = *u*(*t*) − *u*(*t* − 1), as before we set *u*(0) = 0, and, with respect to (11), we have exchanged the order of the sums over *t* and *s* $({\sum }_{t=1}^{T}\,{\sum }_{s=1}^{t}={\sum }_{s=1}^{T}\,{\sum }_{t=s}^{T})$.

This problem can be easily solved with the method of Lagrange multipliers, which brings

12 $${\rm{\Delta }}u(t)=\frac{\sum _{r=t}^{T}\,{\lambda }^{T-r}}{\sum _{s=1}^{T}{(\sum _{r=s}^{T}{\lambda }^{T-r})}^{2}}\ell $$

and

13 $$\begin{array}{rcl}K & = & \frac{{\ell }^{2}}{\sum _{s=1}^{T}{(\sum _{t=s}^{T}{\lambda }^{T-t})}^{2}}\end{array}$$

14 $$\begin{array}{rcl} & = & \frac{{(\lambda -\mathrm{1)}}^{3}\,(\lambda +\mathrm{1)}{\ell }^{2}}{T({\lambda }^{2}-1)+\lambda ({\lambda }^{T}-1)({\lambda }^{T+1}-\lambda -2)}\end{array}\mathrm{.}$$

From this formula we see that, upon increasing the time horizon *T* over which stress propagates, the size of exogenous shocks needed to produce the sought final loss progressively reduces and goes to zero in the limit *T* → ∞. This is expected, as shocks can reverberate over a longer time horizon, and eventually an infinite sequence of infinitesimal shocks can lead to the final loss $\ell$.

However the behavior of the cost function for long time horizons shows the existence of two very distinct regimes: If *λ* > 1 the cost function approaches zero exponentially as $K\sim {\lambda }^{-2T}{\ell }^{2}$, while if *λ* < 1 the cost function decays to zero much more slowly, as $K\sim \frac{{\ell }^{2}{(\lambda -\mathrm{1)}}^{2}}{T}$. The reason of this behavior is that for *λ* > 1 shocks are exponentially amplified by the dynamics.

A similar behavior can be observed for a general matrix of interbank leverages, where the quantity *βλ*_max_, with *λ*_max_ the largest eigenvalue of Λ, now discriminates between the two regimes. We discuss this case in the following section.

### Case study

We discuss an empirical application of the optimization problem (10) to an interbank system representing the largest banks in Europe. We explore the results of this problem of reverse engineering financial contagion as a function of the following variables:

1. The quantity *βλ*_max_, where *λ*_max_ is the largest eigenvalue of the matrix of interbank leverages.
2. The minimal financial loss ${\ell }_{i}$ (in the following we will assume for simplicity that ${\ell }_{i}=\ell$ for all *i*, see the supplementary information for a discussion on the effect of non-homogeneous constraints), which is the target state of the optimized dynamics;
3. The time horizon *T*.

The quantity *βλ*_max_ is the one that determines the stability of the DebtRank dynamics^42^. When *βλ*_max_ < 1 an exogenous shock is progressively dampened by the dynamic, while when *βλ*_max_ > 1 shocks are amplified^32,42^. We note that usually *β* is set to one in the literature on DebtRank^13,37^, while *λ*_max_ depends on the specific pattern of interbank loans and is usually between zero and two. Here we consider only one snapshot of interbank exposures at a given time, so that *λ*_max_ is fixed and equal to 1.38. In order to explore the relevant range of values we therefore vary the parameter *β*.

Because of its interpretation in terms of loss given default, the parameter *β* would normally have an upper bound of one, so that the maximum value for product *βλ*_max_ would be 1.38. In the following we also show results for higher values of *βλ*_max_, corresponding to values of *β* larger than one. The results obtained for *β* > 1 should therefore be interpreted as an exploration of hypothetical scenarios of a higher leveraged system.

#### Aggregate properties of work-case shocks

**Figure 1:**
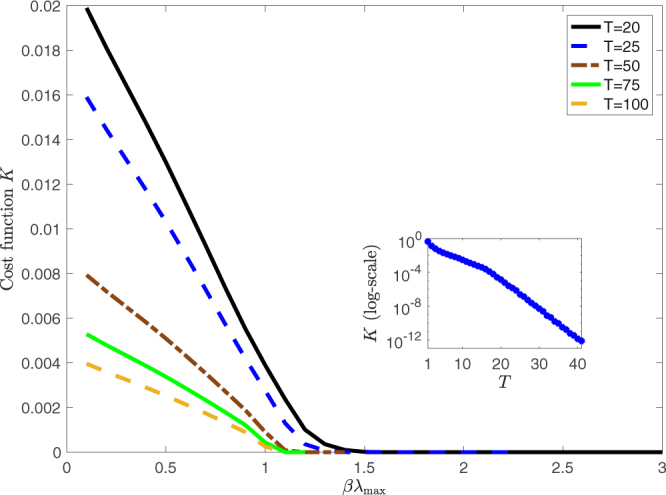
Cost function *K* as a function of β*λ*_max_ for various time horizons *T* and ${\ell }_{i}=\ell =0.1$. Inset: *K* as a function of *T* for *λ*_max_ = 1.5 and ${\ell }_{i}=0.1$. When *βλ*_max_ > 1 worst case shocks decay exponentially fast.

Figure 1 shows the behavior of the cost function *K* as a function of *βλ*_max_ for different time horizons and for ${\ell }_{i}=0.1$ for all *i*. It can be seen that the size of exogenous shocks decreases as a function of *βλ*_max_ across all *T*. As *βλ*_max_ increases the endogenous dynamics of the network lead to a larger amplification of the distress, such that a lower size of external shocks is required to reach the target loss ${\ell }_{i}$.

For a similar reason larger *T* result in smaller *K* independently of the value of *βλ*_max_. The endogenous network dynamics propagate the distress of the previous time step, thereby implying a lower external shock requirement as *T* is increased. Because the iteration map (5) does not reach a fixed point when *βλ*_max_ > 1, even in the absence of external shocks beyond the first time step (i.e. *u*(*t*) = 0 for any *t* > 1), we expect the size of the exogenous shocks *K* to go towards zero exponentially fast in the limit *T* → ∞. This is indeed the case, as shown in the inset of Fig. 1.

**Figure 2:**
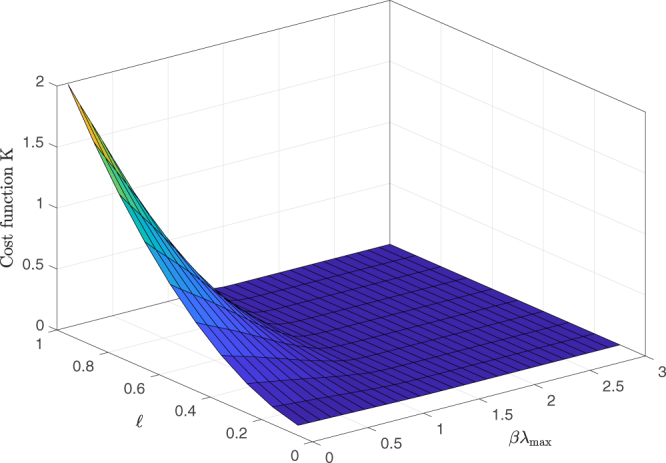
Cost function *K* as a function of target losses ${\ell }_{i}=\ell$ and *βλ*_max_. For large *βλ*_max_ the cost function is independent of the final loss. Results refer to *T* = 20.

The behavior is qualitatively similar for any value of final losses $\ell$, as we show in Fig. 2, where we plot the cost function *K* as a function of *βλ*_max_ and $\ell$ for *T* = 20. From this figure we see that when *βλ*_max_ is large enough the shock needed to cause the sought final losses is relatively independent of $\ell$, while it increases with $\ell$ when *βλ*_max_ < 1.

**Figure 3:**
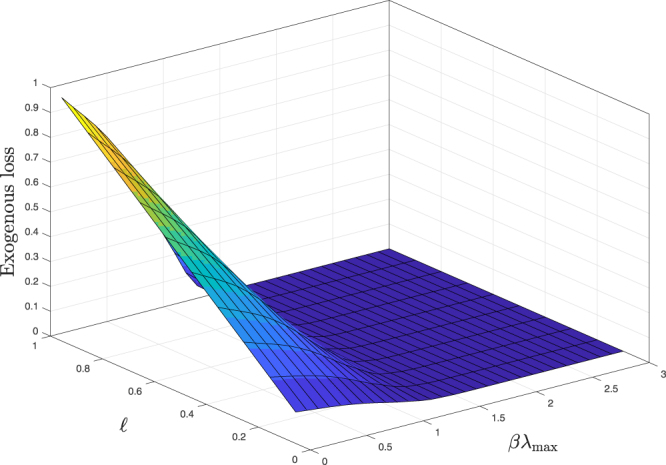
Average exogenous loss per bank ${\sum }_{i}\,{u}_{i}(T)/N$ as a function of the target losses ${\ell }_{i}=\ell$ and *βλ*_max_. For large values of *βλ*_max_ the loss due to the exogenous shock is very small and independent of the final loss. Results refer to *T* = 20.

The cost function is related to the fluctuations over time of the value of external assets along the trajectory of shocks. Another quantity that can be used to characterize the properties of exogenous shocks is ${\sum }_{i}\,{u}_{i}(T)/N$, which is the cumulative exogenous loss (without accounting for any endogenous amplification) due directly to the shock on external assets averaged over banks. This quantity is plotted in Fig. 3 for *T* = 20 as a function of *βλ*_max_ and $\ell$. The behavior is similar to the one discussed for the cost function, but this quantity can be more directly compared with writedowns on banks balance sheet. To provide a frame of reference for a potential shock hitting the equity of banks, we can for instance look at the total writedowns due to the subprime crisis at its height in 2008. We considered Royal Bank of Scotland plc (RBS) and UBS Group AG (UBS), which are both part of our STOXX dataset. In 2008 RBS depreciated its assets by £m 24,000 (depreciation of goodwill and other intangible assets as well as credit losses) and had an equity capital at the beginning of 2008 of £m 91,426^43^, equating to a loss of about 26%. UBS devalued its assets by CHFm 18,240 (difference between depreciation of assets in 2008 and 2007, which is due to writedowns of subprime mortgages) and had an equity capital at the beginning of 2008 of CHFm 43,826^44^, implying a loss of about 42%.

Figure 3 shows the existence of a regime in which an infinitesimal exogenous loss could lead to very large losses due to the endogenous amplification of stress. This result is due to the fact that the DebtRank dynamics becomes unstable for large values of *βλ*_max_^42^, and it is derived under some assumptions: that banks do not react to changing market conditions, that the recovery rate is zero, and that no policy intervention is in place. These are all strong assumptions, and relaxing them would curb the extent of contagion. Nevertheless, the model still provides an upper bound to systemic losses and a simple criterion to discriminate between stable and potentially unstable regions in the space of parameters.

#### Concentration of risk

We have so far looked at the aggregate properties of worst case shocks, however the methodology we propose allows to obtain the distribution of shocks across banks in the system. This information is useful as it enables us to rank banks in terms of their contribution to the aggregate shock, and to identify potential concentrations of vulnerability in the system: If the worst case aggregate shock is uniformly distributed across all banks, then we would expect the system to be more resilient with respect to idiosyncratic failures of individual banks (although the system might be vulnerable with respect to common factors affecting banks portfolios); if the shock is instead highly concentrated in a few banks, the system is vulnerable with respect to the failure of those banks^45^.

**Figure 4:**
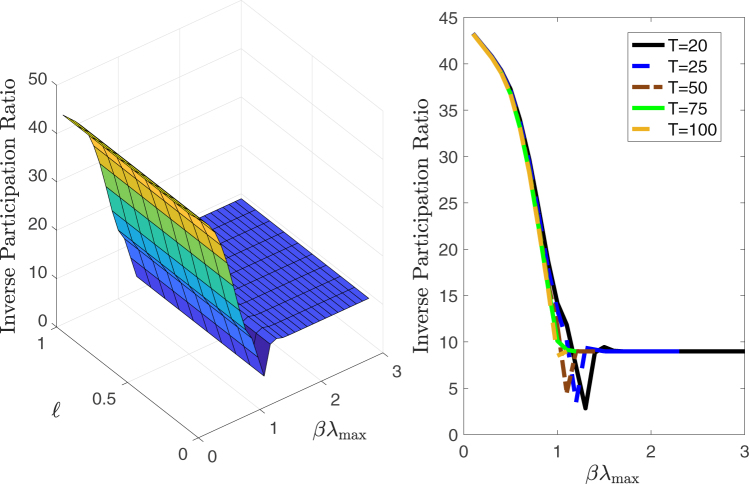
Inverse participation ratio (IPR). Left panel: Inverse participation ratio (IPR) as a function of the target state *ℓ*_*i*_ = *ℓ* and *βλ*_max_ for *T* = 20. Worst case shocks become more concentrated as *βλ*_max_ increases. Right panel: Inverse participation ratio (IPR) as a function of *βλ*_max_ for various time horizons *T* and *ℓ*_*i*_ = 0.1 for all *i*.

The extent of concentration of systemic risk can be quantified by computing the inverse participation ratio (IPR), defined as

15 $${\rm{IPR}}=\frac{1}{\sum _{i=1}^{n}\,{p}_{i}^{2}},$$

where ${p}_{i}=\frac{{K}_{i}}{K}$ for each node *i*. The IPR has a lower bound of 1 when the shock is concentrated in one node, and an upper bound of *n* when the shock is equally spread across all nodes. As it can be seen in Fig. 4a the IPR is unaffected by $\ell$, it decreases significantly as *βλ*_max_ approaches 1, and it becomes constant for *βλ*_max_ > 1.

We have seen that increasing *λ*_max_ leads to a reduction of the aggregate size of the shock *K* needed to drive the system towards a certain loss and to a concentration of shocks upon a smaller set of banks. We stress here that these two behaviors have different roots. The reduction of *K* is due to the fact that the system becomes more unstable as leverage increases. The concentration of risk is due to the heterogeneity of leverage across banks. In fact, in the homogeneous system considered above this concentration does not occur. The heterogeneity of banks’ leverages, shown in Supplementary Fig. 1, suggests that some banks have lent significantly more than others relative to their equity capital. Some banks therefore contribute to the risk of the system much more than other banks, which leads to the concentration of shocks observed in Fig. 4a.

The observed concentration of risk as measured by the IPR is one of the most significant findings of this study. It indicates that as the pace of the dynamic in an interbank market increases, a decreasing number of nodes in the system can cause systemic events. In other words, while for small *βλ*_max_ < 1 a large fraction of nodes need to experience stress in order for the system to observe significant losses, when *βλ*_max_ > 1 only a few nodes are sufficient to cause significant losses. In the next section we will use this insight to devise a policy experiment to reduce the observed system losses.

#### A simple policy experiment

The trajectories of worst case shocks that we have computed correspond to the least extreme scenario that leads to a prescribed final loss equal or greater than $\ell$. In this sense, the concentration of shocks discussed above suggests that there is a regime (high *βλ*_max_) where systemic vulnerabilities can be associated with a small set of banks, those where the aggregate shock is concentrated.

To show that this is the case we run the dynamics (5) forward applying the worst case shocks to a subset of the nodes. We then compute the final loss observed in the system divided by the final loss observed when all banks are stressed and plot this ratio as a function of the fraction of stressed banks.

**Figure 5:**
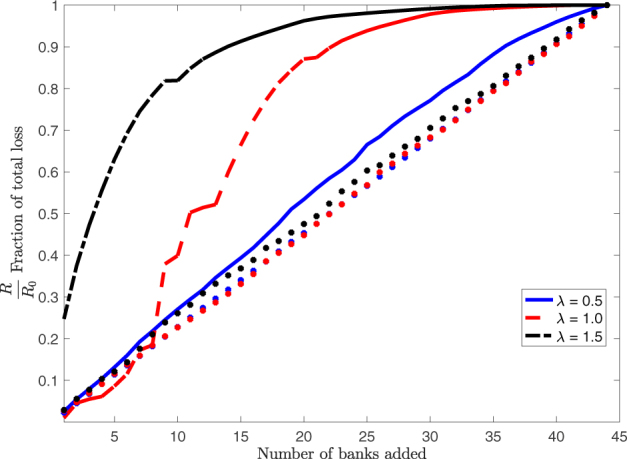
Incremental addition of *u*(*t*) to banks, shown on x-axis, in a decreasing order of the size of their individual shocks *K*_*i*_ for various *βλ*_max_. The fraction of total loss is shown as a function of the fraction of total losses caused by the shocks *u*_*i*_(*t*) on the y-axis. The stars represent the average across 500 simulations in which banks were randomly selected (same colors as for the three lines corresponding to the three different *β* *λ*_max_ shown in the figure legend, where *βλ*_max_ is denoted as λ). Results shown for *T* = 20 and ${\ell }_{i}=0.1$ for all *i*. For *β* *λ*_max_ = 1.5 the shock on the first five banks already accounts for 70% of final losses.

Figure 5 shows the result of this experiment for different values of *β* *λ*_max_ when the stressed banks are those with the highest values of *K*_*i*_. We also report the results for the benchmark case in which stressed banks are randomly selected. As expected, deviations from the benchmark case become larger as the system becomes more unstable. The concentration of systemic risk in the system can be seen particularly when *β* *λ*_max_ = 1.5 (black line), in which case the exogenous shock of 5 banks can lead to roughly 70% of all observed final losses. Note that when banks are randomly selected then no such concentration is observed (stars in equivalent colors in the figure).

These insights can be used for a policy experiment on the equity capital requirements of individual banks, that aims to reduce the observed financial losses under the scenario identified through the reverse stress test. In fact the contribution of each bank to the aggregate shock can be used to rank banks in terms of their systemic impact.

**Figure 6:**
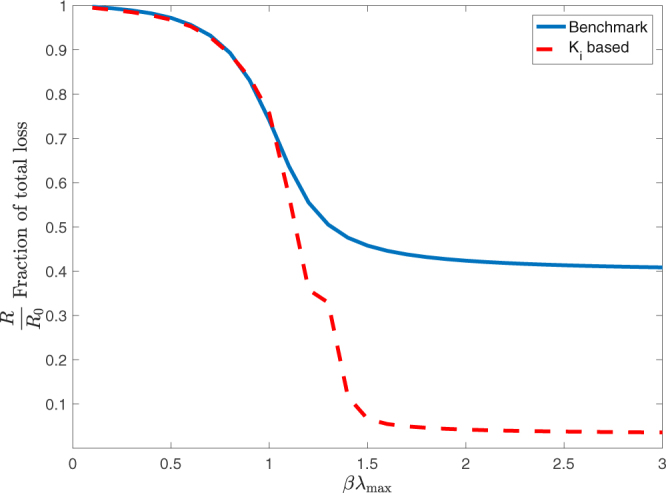
Comparison of different policies to reduce the observed total financial losses. In both cases the same amount of money was allocated in different manners to the equity of each bank. Losses *R* are recomputed after the equity was increased and expressed as a fraction of the original losses *R*_0_ on the y-axis. These results are shown as a function of *βλ*_max_ along the x-axis. When *βλ*_max_ < 1 the benchmark is slightly more effective than the *K*_*i*_ based policy, however when *βλ*_max_ > 1 then the policy based on the relative size of each banks’ shock *K*_*i*_ is significantly more effective. Results shown for *T* = 20 and ${\ell }_{i}=0.1$ for all *i*.

The results of a policy exercise in which capital is allocated depending on this ranking are shown in Fig. 6. Specifically, we consider a situation in which we increase the total capital in the system by 5% and a policy by which such capital is spread across banks proportionally to the size of the shock computed from the reverse stress test, i.e. bank *i* receives a proportion *K*_*i*_/*K* of the total additional capital. We then compare this policy with a benchmark according to which the equity of each bank is increased by 5% of its current value. This benchmark mimics the case of a homogeneous (relative) increase in the capital requirement of banks. For both policies, we compute the total relative losses $R={\sum }_{i}^{n}\,{h}_{i}(T)$ under the scenario identified through the reverse stress test and compare it with the total losses observed in absence of policy intervention *R*_0_.

Currently the Basel III capital regulations stipulate a minimum level of equity capital of 4.5% plus an additional conservation buffer 2.5% of a bank’s total risk-weighted assets. Moreover national regulators may require an addition capital increase of up to 2.5% if an increase in risk is observed across the system^46^. Put together these requirements are referred to as the Common Equity Tier 1 ratio. The definition of risk-weighted assets is thus a crucial factor in determining the level of equity capital. In brief, each asset held by a bank is given a factor based on its riskiness, which scales its weight in the aggregation of total assets. Our policy approach of increasing the equity capital is thus different to current regulations. The focus of this section is to identify whether this difference is warranted, by testing whether our policy is effective in stabilising the system. The European Banking Authority (EBA) reported after its 2016 stress test exercise of the 51 largest banking institutions in Europe, that the average Common Equity Tier 1 ratio increased from 8.9% at the end of 2010 to 13.2% at the end of 2015. This corresponds to an increase of about 50% in equity capital across these banks^47^. In this respect our suggested 5% capital increase is quite small. The fact that such small increase of equity can lead to a significant reduction of losses is due to the linear assumption of DebtRank. We note however that our focus here is not on the absolute performance of our policy, but rather on its performance relative to the benchmark. We also note that there is a large overlap between the banks involved in the stress tests of the EBA and the STOXX index used in this paper. In fact 10 out of the 15 European banks classified as global systemically important banks in 2015 are in the top 15 of our ranking of banks based on their systemic importance *K*_*i*_^48^.

As it can be seen in Fig. 6 when *βλ*_max_ < 1 the two policies achieve a similar reduction of total losses, while the policy based on the reverse stress test becomes more effective when *βλ*_max_ > 1. The reason for this result is that the explosive dynamics when *βλ*_max_ > 1 lead to a concentration of systemic risk in a few banks on which an effective policy should concentrate.

**Figure 7:**
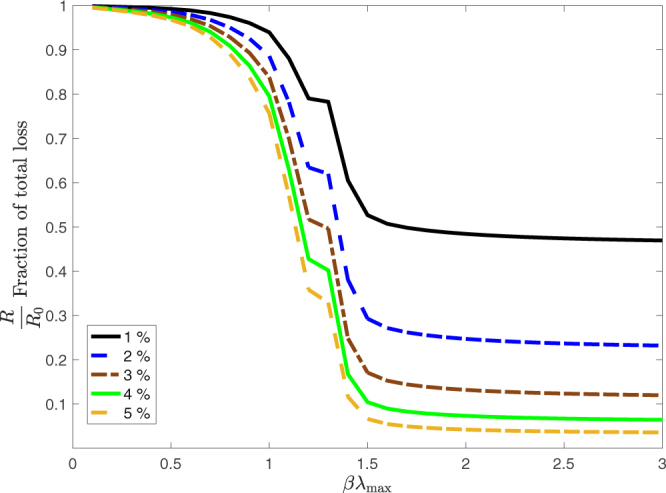
Loss reduction for the policy based on our ranking as a function of *βλ*_max_ for different amounts of capital injected into the system, i.e. the percentage in the legend indicates the percentage increase of the total equity capital of the system. The effectiveness of increasing capital has rapidly vanishing returns of scale. Results shown for *T* = 20 and ${\ell }_{i}=0.1$ for all *i*.

Figure 7 shows that larger amounts of additional capital allocated to the equity base of each bank result in a further reduction of the observed losses. The figure shows this for the policy based on nodal shocks. Note however that the impact of an increased equity allocation has decreasing returns of scale. The impact of an increase from 1% to 2% is much larger than the impact of 4% relative to that of 5%. This behavior occurs for the benchmark policy as well, but in that case it is not as pronounced. This is due to the fact that the policy based on the size of shocks is much more effective in allocating the additional equity as compared to the benchmark.

The decreasing returns of scale of our policy are of high relevance to financial regulators. Higher capital requirements come at large costs to banks, not only due to the costs involved in raising the additional capital but also in terms of lower returns. The fact that equity capital requirements beyond a certain level lead to drastically diminished marginal reductions in risk, points towards an optimal trade-off between the costs of increasing equity capital requirements and their effectiveness in reducing risk.

#### Robustness of results

To test the robustness of our results we collected data of the previous year (2014) and following year (2016) to compare to the simulations undertaken in this paper, which are based on 2015 data. The results are very similar across the years (see Supplementary Information). The cost function *K* and its related measures such as the IPR display the same behavior across the three years. Furthermore the ranking of nodes is very robust across time, such that the results of the equity capital policy are very similar in 2014 and 2016 compared to those of 2015 presented here. The correlation between nodal shocks across different years is always larger than 0.9. The strong similarity between the years is due to the relative stability of equity, interbank assets and liabilities of individual banks.

We additionally tested the influence of our choice to study only the complete STOXX network on our results. We therefore redid the entire analysis presented in the paper by using the RAS algorithm to construct a network with density of 60%. We found the behavior of the total shock *K*, the IPR and the policy very similar. We show the corresponding plots in the supplementary information, together with a characterization of the cost function and the IPR as a function of the network density.

## Discussion

We have introduced a simple reverse stress testing methodology to reverse engineer distress contagion in financial networks. We reversed the standard stress testing approach by setting a specific outcome, the loss of a certain fraction of the equity of each bank, and looking for the scenario with smallest shocks that could lead to such outcome over a given time horizon.

We considered a system of interbank relationships based on 2015 annual data of the equity, interbank lending and borrowing of the largest 44 stock exchange listed European banks. We found that at the aggregate level the size of the worst case shock decreases as the largest eigenvalue *λ*_max_ increases, but that at the same time the shock gets concentrated in a smaller number of banks. On the basis on this concentration of worst case shocks, we ranked banks in terms of their systemic impact. Based on this ranking we suggested a simple policy of capital allocations that significantly reduces the vulnerability of the system with respect to the identified scenario in the regime of high endogenous amplification.

Our analysis can be improved in several directions: First of all, we considered a simple linear dynamical rule of distress propagation. Although common in the literature of financial contagion, this assumption can at best be considered only an approximation of the true dynamics. In a more general case, it is still possible to write an optimization problem analogous to (10) to perform the reverse stress test. The main difference with respect to the case here considered would be the presence of a non-linear constraint, but the optimization problem could still be solved numerically. Secondly, we here only considered the DebtRank algorithm as a means of stress propagation in interbank networks. Other important channels of contagion operate in interbank networks, without which a complete understanding of the propagation of risk in a network is not possible^36^. Two specific contagion channels are overlapping portfolio risk^35^ and liquidity risk, that may result in fire-sales^49,50^. Extending the reverse stress testing framework to these types of contagion is left for future work. Indeed further studies of different channels of contagion should also take into account the specific characteristics of individual institutions that play a large role in the concentration of risk^34^. A third limitation of our analysis is the fact that we only considered direct long exposures between banks. Banks interact in many ways in the real world, and a more realistic scenario would consider a multilayer description of the network of interbank interactions. In this respect, our present analysis corresponds to an aggregation of the multilayer structure into a single layer^51–53^. However, it would be important to look also at the disaggregated multilayer structure because the properties of aggregated and non-aggregated systems have been shown to differ in some cases^52^. Fourth, we considered the case of banks as passive investors. This is certainly a useful benchmark, but a more realistic scenario would also account for the reaction of banks to changing market conditions.

In spite of all the present limitations, our analysis suggests that reverse stress testing is a useful tool for the identification of vulnerabilities at the systemic level, and we believe this is an interesting avenue of future investigation with potentially relevant policy implications.

## Methods

### Data

We collected data of the 44 banks belonging to the STOXX Europe 600 Banks index, ticker symbol: SX7P. In particular, for each bank we collected information on its equity, total interbank assets (advances and loans to banks) and total interbank liabilities (deposits due to other banks) for the entire year of 2015. Note that we converted all values to Euros using the prevailing foreign exchange rates on November 11th 2016. We then used the RAS algorithm to reconstruct a matrix of interbank liabilities to represent an interbank lending network. Starting from total interbank assets and liabilities of each bank, the RAS algorithm allows an allocation of interbank loans across counterparties^30^. If no further constraints are added, the outcome of the RAS algorithm is a complete weighted network of interbank claims. Although real interbank networks are far from complete^54–57^, here for simplicity we focus on this limiting case which allows us to focus on the mechanics of reverse stress testing only, rather than on the interplay between network topology and contagion. In order to test how much our results are driven by the fact that the network we consider is complete, we have replicated our analysis for an incomplete network with density 60%. The results of this analysis are reported in the Supplementary Information, section 1, and are in agreement with those obtained for the complete network.

## Electronic supplementary material

Supplementary information

## References

[1] May RM, Arinaminpathy N (2010). Systemic risk: the dynamics of model banking systems. Journal of the Royal Society Interface.

[2] Haldane AG, May RM (2011). Systemic risk in banking ecosystems. Nature.

[3] Glasserman P, Young HP (2016). Contagion in financial networks. Journal of Economic Literature.

[4] Allen F, Gale D (2000). Financial contagion. Journal of political economy.

[5] Eisenberg L, Noe TH (2001). Systemic risk in financial systems. Management Science.

[6] Birch A, Aste T (2014). Systemic losses due to counterparty risk in a stylized banking system. Journal of Statistical Physics.

[7] Nier E, Yang J, Yorulmazer T, Alentorn A (2007). Network models and financial stability. Journal of Economic Dynamics and Control.

[8] Gai, P. & Kapadia, S. Contagion in financial networks. In *Proceedings of the Royal Society of London A*: *Mathematical*, *Physical and Engineering Sciences*, rspa20090410 (The Royal Society, 2010).

[9] Drehmann M, Tarashev N (2013). Measuring the systemic importance of interconnected banks. Journal of Financial Intermediation.

[10] Iori G, Jafarey S, Padilla FG (2006). Systemic risk on the interbank market. Journal of Economic Behavior & Organization.

[11] Martinez-Jaramillo S, Alexandrova-Kabadjova B, Bravo-Benitez B, Solórzano-Margain JP (2014). An empirical study of the mexican banking system’s network and its implications for systemic risk. Journal of Economic Dynamics and Control.

[12] Iori G, De Masi G, Precup OV, Gabbi G, Caldarelli G (2008). A network analysis of the italian overnight money market. Journal of Economic Dynamics and Control.

[13] Battiston S, Puliga M, Kaushik R, Tasca P, Caldarelli G (2012). Debtrank: Too central to fail? Financial networks, the fed and systemic risk. Scientific reports.

[14] Lenzu S, Tedeschi G (2012). Systemic risk on different interbank network topologies. Physica A: Statistical Mechanics and its Applications.

[15] Caccioli F, Catanach TA, Farmer JD (2012). Heterogeneity, correlations and financial contagion. Advances in Complex Systems.

[16] Tedeschi G, Mazloumian A, Gallegati M, Helbing D (2012). Bankruptcy cascades in interbank markets. PloS one.

[17] Roukny T, Bersini H, Pirotte H, Caldarelli G, Battiston S (2013). Default cascades in complex networks: Topology and systemic risk. Scientific reports.

[18] Georg C-P (2013). The effect of the interbank network structure on contagion and common shocks. Journal of Banking & Finance.

[19] Caccioli F, Shrestha M, Moore C, Farmer JD (2014). Stability analysis of financial contagion due to overlapping portfolios. Journal of Banking & Finance.

[20] Cimini, G., Squartini, T., Garlaschelli, D. & Gabrielli, A. Systemic risk analysis on reconstructed economic and financial networks. *Scientific Reports* **5** (2015). 10.1038/srep15758 PMC4623768 26507849

[21] Battiston S, Caldarelli G, D’Errico M, Gurciullo S (2016). Leveraging the network: a stress-test framework based on debtrank. Statistics & Risk Modeling.

[22] Petrone, D. & Latora, V. A hybrid approach to assess systemic risk in financial networks. *arXiv preprint arXiv*:*1610*.*00795* (2016).

[23] Duarte, F. & Eisenbach, T. M. Fire-sale spillovers and systemic risk. *FRB of New York Staff**Report No*. *645* (2015).

[24] Cont, R. & Schaanning, E. F. Fire sales, indirect contagion and systemic stress-testing. *SSRN*: *2541114* (2016).

[25] Amini H, Cont R, Minca A (2012). Stress testing the resilience of financial networks. International Journal of Theoretical and Applied Finance.

[26] Barucca, P. *et al*. Network valuation in financial systems. *SSRN*: *2795583* (2016).

[27] Grundke, P. & Pliszka, K. A macroeconomic reverse stress test. *Bundesbank Discussion**Paper No*. *30*/*2015* (2015).

[28] Flood MD, Korenko GG (2015). Systematic scenario selection: stress testing and the nature of uncertainty. Quantitative Finance.

[29] McNeil AJ, Smith AD (2012). Multivariate stress scenarios and solvency. Insurance: Mathematics and Economics.

[30] Bacharach M (1965). Estimating nonnegative matrices from marginal data. International Economic Review.

[31] Bardoscia M, Caccioli F, Perotti JI, Vivaldo G, Caldarelli G (2016). Distress propagation in complex networks: the case of non-linear debtrank. PloS one.

[32] Bardoscia M, Battiston S, Caccioli F, Caldarelli G (2017). Pathways towards instability in financial networks. Nature Communications.

[33] Upper C (2011). Simulation methods to assess the danger of contagion in interbank markets. Journal of Financial Stability.

[34] Glasserman P, Young HP (2015). How likely is contagion in financial networks?. Journal of Banking & Finance.

[35] Caccioli F, Farmer JD, Foti N, Rockmore D (2015). Overlapping portfolios, contagion, and financial stability. Journal of Economic Dynamics and Control.

[36] Clerc, L. *et al*. Indirect contagion: the policy problem. *European Systemic Risk Board Occasional Paper Series* January (2016).

[37] Battiston S, DErrico M, Gurciullo S (2016). Debtrank and the network of leverage. The Journal of Alternative Investments.

[38] Visentin, G., Battiston, S. & D’Errico, M. Rethinking financial contagion (2016).

[39] Liu Y-Y, Slotine J-J, Barabási A-L (2011). Controllability of complex networks. Nature.

[40] Cimini G, Serri M (2016). Entangling credit and funding shocks in interbank markets. PloS one.

[41] Amini H, Cont R, Minca A (2016). Resilience to contagion in financial networks. Mathematical Finance.

[42] Bardoscia M, Battiston S, Caccioli F, Caldarelli G (2015). Debtrank: A microscopic foundation for shock propagation. PloS one.

[43] RBS. Group Annual Report for 2008. http://investors.rbs.com/reports-archive/archived.aspx (2009).

[44] UBS. Annual Report 2008 (restated 20 May 2009). https://www.ubs.com/global/en/about_ubs/investor_relations/annualreporting/archive.html (2009).

[45] Albert R, Jeong H, Barabasi A-L (2000). Error and attack tolerance of complex networks. Nature.

[46] Basel Committee On Banking Supervision. *Basel III*: *A global regulatory framework for more resilient banks and banking systems* (2011).

[47] European Banking Authority. 2016 EU-Wide Stress Test (29 July 2016) (2016).

[48] Financial Stability Board. 2015 Update of List of Global Systemically Important Banks (2015).

[49] Battiston, S., Delli Gatti, D., Gallegati, M., Greenwald, B. & Stiglitz, J. E. Liaisons dangereuses: Increasing connectivity, risk sharing, and systemic risk. *Journal of Economic Dynamics and Control* **36**, 1121–1141, 10.1016/j.jedc.2012.04.001, arXiv:1011.1669v3 (2012).

[50] Cifuentes R, Ferrucci G, Shin HS (2005). Liquidity Risk and Contagion. Journal of the European Economic Association.

[51] Poledna S, Molina-Borboa JL, van der Leij M, Martinez-Jaramillo S, Thurner S (2015). Multi-layer network nature of systemic risk in financial networks and its implications. J Financ Stab.

[52] Hackett A, Cellai D, Gómez S, Arenas A, Gleeson JP (2016). Bond percolation on multiplex networks. Physical Review X.

[53] Langfield S, Liu Z, Ota T (2014). Mapping the uk interbank system. Journal of Banking & Finance.

[54] Boss M, Elsinger H, Summer M, Thurner S (2004). Network topology of the interbank market. Quantitative Finance.

[55] Squartini, T., van Lelyveld, I. & Garlaschelli, D. Early-warning signals of topological collapse in interbank networks. *arXiv preprint arXiv*:*1302*.*2063* (2013). 10.1038/srep03357 PMC3842548 24285089

[56] Finger K, Fricke D, Lux T (2013). Network analysis of the e-mid overnight money market: the informational value of different aggregation levels for intrinsic dynamic processes. Computational Management Science.

[57] Fricke, D., Finger, K., Lux, T. *et al*. On assortative and disassortative mixing in scale-free networks: The case of interbank credit networks. Tech. Rep., Kiel Working Paper (2013).

