# Supplementary material for: Reverse stress testing interbank networks
Source: Sci Rep. 2017 Nov 15;7:15616. doi: 10.1038/s41598-017-14470-1 (PMC5688116; doi:10.1038/s41598-017-14470-1)
Supplement: Supplementary file 1 — Supplementary information [file 41598_2017_14470_MOESM1_ESM.pdf]

Supplementary Information to: Reverse stress testing interbank networks

Daniel Grigat and Fabio Caccioli

Distribution of interbank leverages

We show in fig. 1 the distribution of the interbank leverage of each of the $n = 44$ banks studied as part of the STOXX network in 2015. The interbank leverage of a bank is the ratio between its total interbank assets and its equity. The interbank leverage of bank i is therefore defined as $\sum_j \Lambda_{ij}$, where Λ_{ij} is the matrix of interbank leverages defined as $\Lambda_{ij} = A_{ij}/E_i$. A_{ij} denotes the value of the exposure of bank i to bank j, and E_i denotes the equity of bank i. It can be seen in fig. 1 that there is some heterogeneity in the distribution of interbank leverage across banks. Most banks have an interbank leverage smaller than one, however one bank has lent more than 6 times the amount of its equity.

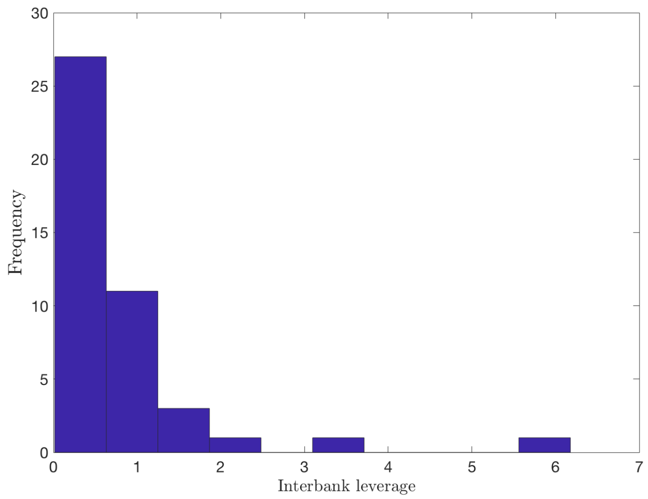

Supplementary Figure 1: Histogram of interbank leverages of each bank in the STOXX network under investigation. Banks have heterogeneous strategies in terms of their interbank leverage.

The following table presents some basic properties of interbank leverages in the data.

year	minimum value	maximum value	mean	standard deviation
2014	0.005	6.6	1.1	1.2
2015	0.017	6.2	0.8	1.0
2016	0.18	6.0	0.9	1.0

Effect of network density

In this section we look at the behavior of the worst case shocks as a function of the network density.

The networks have been reconstructed through a two-stage method that has been previously used in the literature, see for instance [3]. First, we constructed a binary adjacency matrix using the fitness model introduced in [4], then we applied the RAS algorithm to the binary network thus obtained [2]. We note that alternative procedures of network reconstruction, which might lead to different outcomes for what concerns the structure of the network, could be used instead of the fitness model here considered (see [1] for a recent review).

The data presented below refer to averages over 100 networks generated with the fitness model. All other aspects of the network remained the same as in the main paper, which we described in the methodology section. In the main paper we studied the complete version of the network. We undertook this analysis to test whether the assumption of complete network used in the main text has a strong effect on the outcome of the reverse stress test framework. We find that the main results are indeed stable.

First we reproduce the results of the main paper for the STOXX network with a density of 60%, then we discuss the behavior of the cost function and the inverse participation ratio as a function of the density.

Figure 2 shows the average exogenous loss per bank as a function of the target losses ℓ and $\beta\lambda_{\max}$ for networks with density 60%.

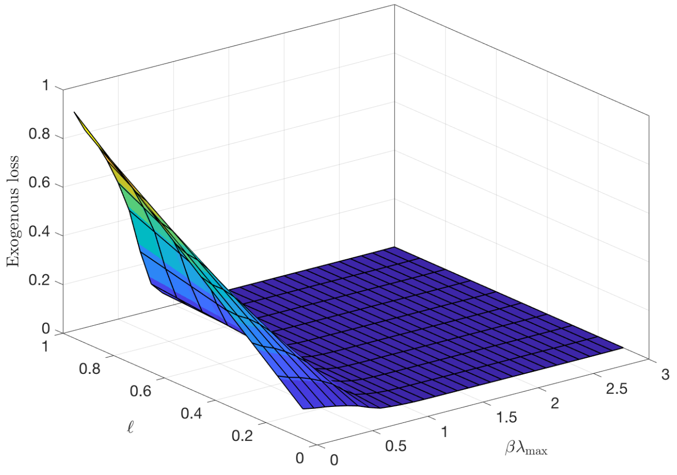

Supplementary Figure 2: Average loss per bank $\sum_i u_i(T)/N$ as a function of the target losses $\ell_i = \ell$ and $\beta\lambda_{\max}$. For large $\beta\lambda_{\max}$ the cost function is independent of the final loss. Results refer to $T = 20$.

Figure 3 shows the Inverse Participation Ratio (IPR) for networks with density 60% and the corresponding concentration of risk on a smaller number of banks as $\beta\lambda_{\max}$ is increased.

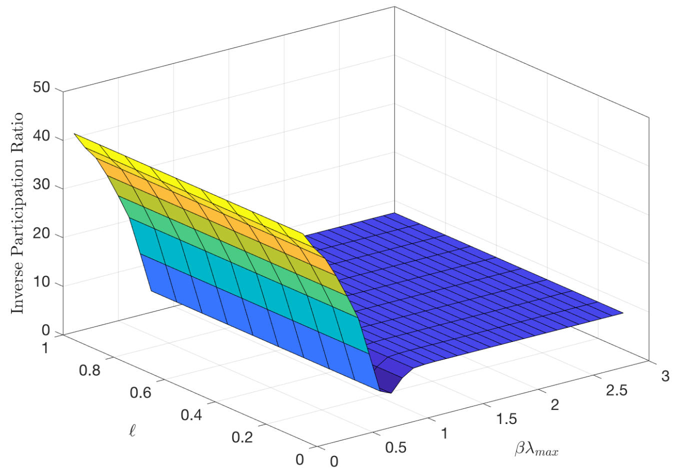

Supplementary Figure 3: Inverse participation ratio (IPR) as a function of the target state ℓ_i and $\beta\lambda_{\max}$ for $T = 20$. The exogenous shocks become more concentrated as $\beta\lambda_{\max}$ increases.

Figure 4 and fig. 5 show the result of the policy experiment performed on networks with density 60%,. The benchmark policy increases the equity of each bank by 5% whereas the K_i-based policy allocates an increase in the total equity of the system by 5% according to banks' decreasing systemic importance, that we defined as $\frac{K_i}{K}$. It can be seen in fig. 4 that, as for the complete network shown in the main paper in figure 6, the policy based on the ranking obtained from the reverse stress testing is much more effective relative to the benchmark policy as the dynamic becomes faster and less stable, i.e. as $\beta\lambda_{\max}$ increases.

Finally, fig. 5 shows the decreasing returns of scale of the K_i-based policy as the total equity of the system is increased. This result is very similar to that of the full STOXX network shown in the main paper in figure 7, and indicates a trade-off between banks' additional costs in increasing their equity capital against the ability of additional equity capital to reduce the observed systemic losses.

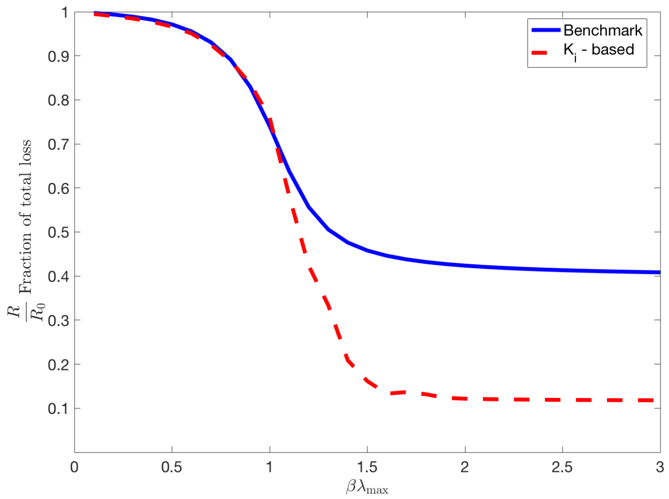

Supplementary Figure 4: Comparison of different policies to reduce the observed total financial losses. In both cases the same amount of money was allocated in different manners to the equity of each bank. Losses R are recomputed after the equity was increased and expressed as a fraction of the original losses R_0 on the y-axis. When $\beta\lambda_{\max} < 1$ the benchmark is slightly more effective than the K_i based policy, however when $\beta\lambda_{\max} > 1$ then the policy based on the relative size of each banks' shock K_i is significantly more effective. Results refer to $T = 20$ and $\ell_i = 0.1$ for all i.

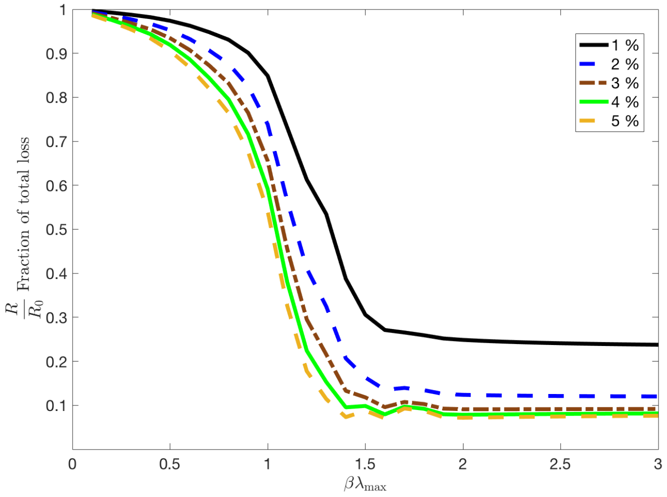

Supplementary Figure 5: Loss reduction for the policy based on our ranking of K_i as a function of $\beta\lambda_{\max}$ for different amounts of capital injected into the system, i.e. the percentage in the legend indicates the increase of the total equity capital of the system. The effectiveness of increasing capital has rapidly vanishing returns of scale. Results refer to $T = 20$ and $\ell_i = 0.1$ for all i.

Finally, fig. 6 and fig. 7 show the behavior of the cost function and the inverse participation ratio as a function of the network density for three values of $\beta\lambda_{\max}$. We see from the figures that there is some dependency of the two quantities on the density of the network, but that this dependency appears to be smooth. In particular we see that the main properties that we have highlighted in the main text, namely that the cost function and the inverse participation ratio decrease as $\beta\lambda_{\max}$ increases, are preserved across all values of density we explored.

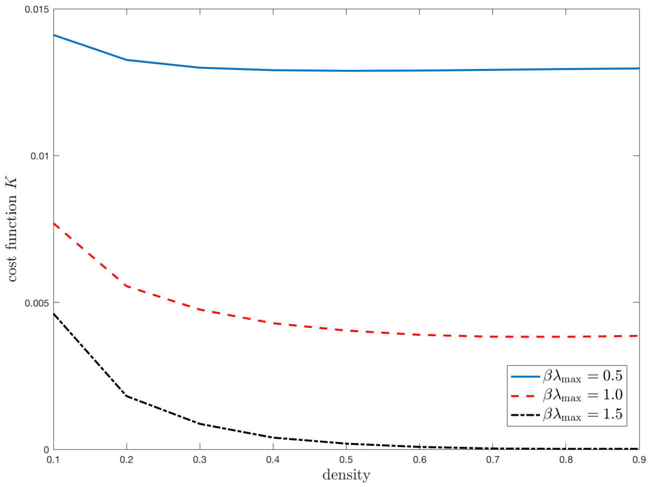

Supplementary Figure 6: Cost function as a function of network density for three values of $\beta\lambda_{\max}$. Results refer to $T = 20$ and $\ell_i = 0.1$ for all i. Higher values of $\beta\lambda_{\max}$ are associated with lower values of the cost function for all densities.

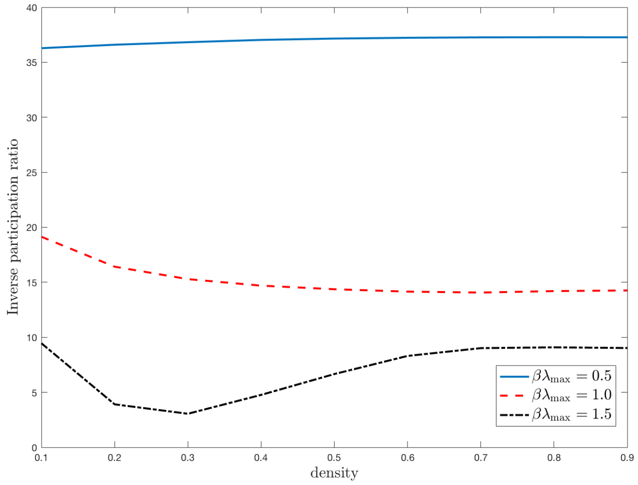

Supplementary Figure 7: Inverse participation ratio as a function of network density for three values of $\beta\lambda_{\max}$. Results refer to $T = 20$ and $\ell_i = 0.1$ for all i. Higher values of $\beta\lambda_{\max}$ are associated with lower values of the inverse participation ratio for all densities.

Results for 2014 and 2016

As a further robustness check, in this section we report the results we obtained for the years 2014 and 2016. Figure 8 shows the cost function as a function of the target losses ℓ (assumed to be the same across all banks) and $\beta\lambda_{\text{max}}$. The figure is analogous to figure 2 of the main paper, and it shows a similar behavior of the cost function. In particular we see that for all values of ℓ the cost function becomes smaller as $\beta\lambda_{\text{max}}$ increases, and that for values of $\beta\lambda_{\text{max}}$ larger than one the cost function depends very weakly on the target loss.

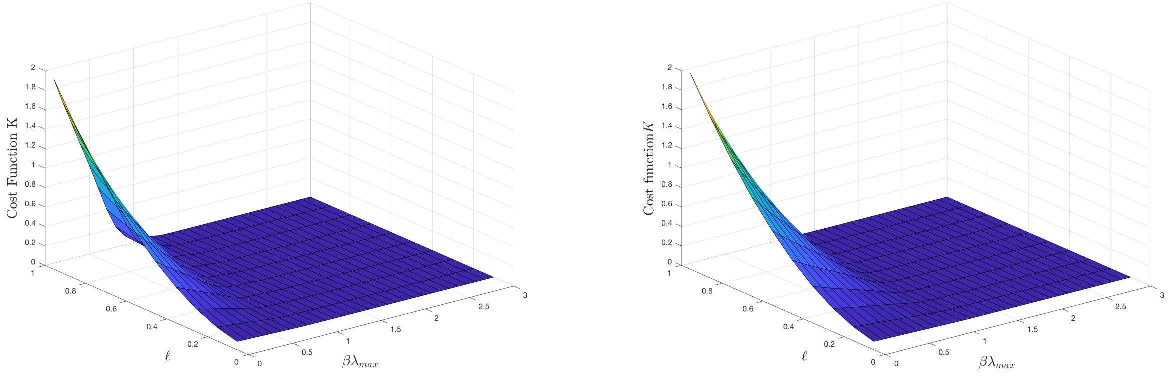

Supplementary Figure 8: Cost function as a function of the target losses $\ell_i = \ell$ and $\beta\lambda_{\text{max}}$ for the years 2014 (left panel) and 2016 (right panel). Results refer to $T = 20$.

A similar behavior is observed for the average exogenous loss (that is the loss of equity due to direct losses on external assets) as a function of ℓ and $\beta\lambda_{\text{max}}$, shown in Figure 9, which replicates the analysis of figure 3 in the main paper.

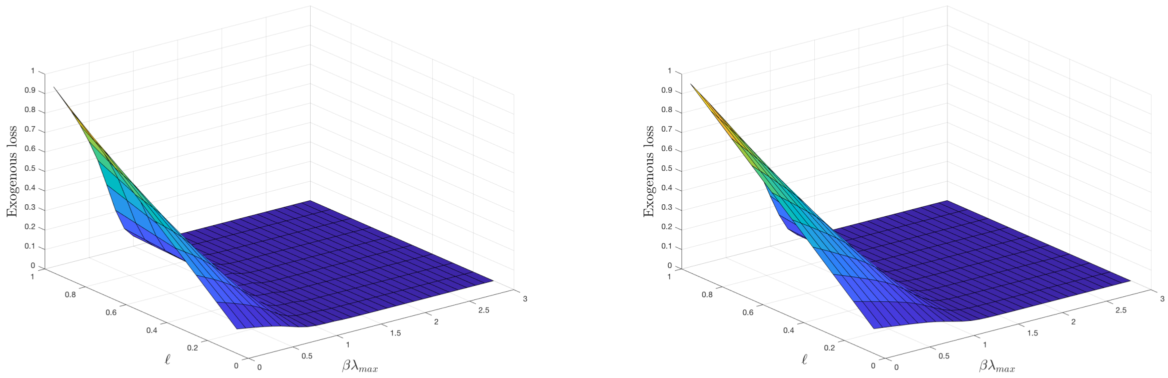

Supplementary Figure 9: Average exogenous loss per bank $\sum_i u_i(T)/N$ as a function of the target losses $\ell_i = \ell$ and $\beta\lambda_{\max}$ for the years 2014 (left panel) and 2016 (right panel). Results refer to $T = 20$.

Figure 10 shows instead the behavior of the inverse participation ratio of the vector of shocks computed as a result of the optimization problem that defines the reverse stress test protocol. This figure is similar to figure 4a of the main paper, and it conveys the same message: For small values of $\beta\lambda_{\max}$, the worst case shock is more or less equally spread across banks, but upon increasing $\beta\lambda_{\max}$ it becomes more concentrated on a subset of banks.

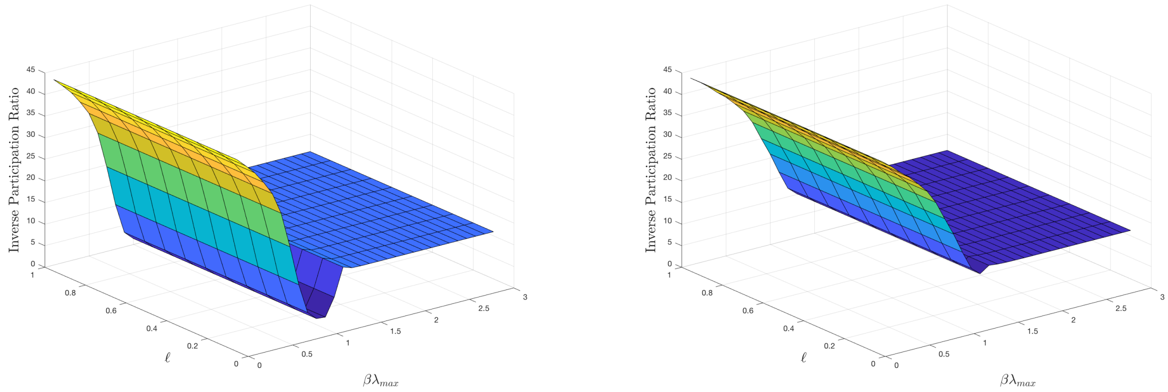

Supplementary Figure 10: Inverse participation ratio as a function of the target losses $\ell_i = \ell$ and $\beta\lambda_{\max}$ for the years 2014 (left panel) and 2016 (right panel). Results refer to $T = 20$.

Finally, fig. 11 shows the outcome of the same policy experiment shown in figure 6 of the main paper. As for 2015, we observe that the policy intervention based on the outcome

of the reverse stress testing is more effective than the benchmark.

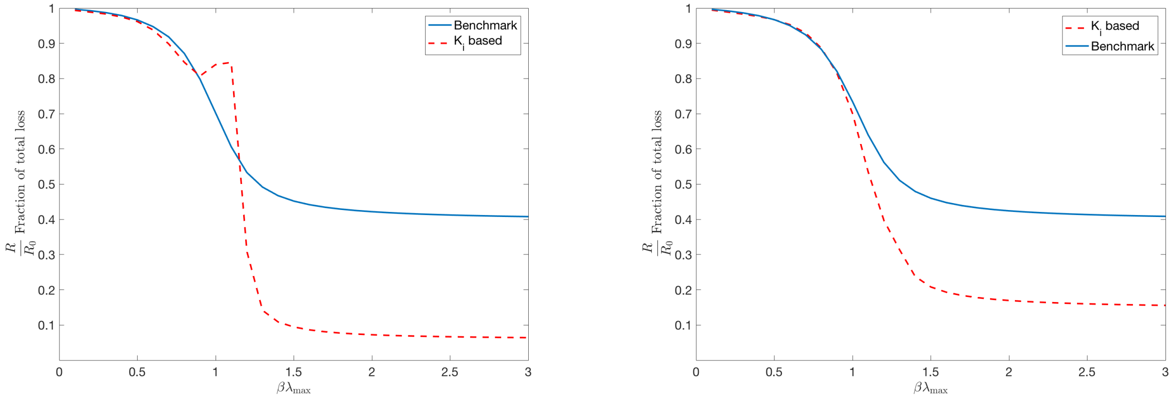

Supplementary Figure 11: Comparison between the benchmark policy and the one based on the reverse stress testing for the years 2014 (left panel) and 2016 (right panel). Results refer to $T = 20$.

Effect of non-homogeneous constraints

Although the reverse stress test methodology introduced in the paper is more general, we have so far always considered the situation in which the constraints on the final losses are homogeneous across banks, i,.e. $\ell_i = \ell$ for all i. Here we relax this assumption and we consider the situation in which ℓ_i is taken from a uniform distribution between zero and one, and we replicate the analysis performed in the main text for the case of homogeneous constraints with $\ell_i = 0.5$ for all banks (we choose 0.5 so that the average loss is the same for uniform and non-uniform constraints). We report the results of our analysis in fig. 12, fig. 13 and fig. 14. We see from the figures that the qualitative properties we highlighted for the homogeneous case are present also in the non-homogeneous one. In particular, the existence of two regimes for low and high values of $\beta\lambda_{\max}$, the concentration of shocks in a smaller number of banks as $\beta\lambda_{\max}$ increases, and the higher effectiveness of the policy based on the outcome of the reverse stress test compared to the benchmark.

Some differences can also be observed in the figures between the homogeneous and non-homogeneous cases. In particular we see that the cost function is overall slightly higher and that shocks appear to be more concentrated in presence of non-homogeneous constraints of the form here considered. The policy based on the ranking obtained from the reverse stress testing also appears to be more effective in the case of non-homogeneous constraints.

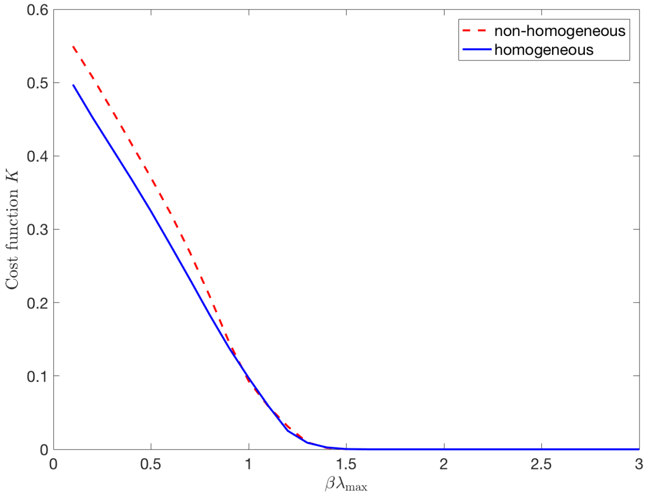

Supplementary Figure 12: Cost function as a function of $\beta\lambda_{\max}$: comparison between the case where $\ell_i = 0.5$ for all nodes vs. the case where ℓ_i is drawn from a uniform distribution between zero and one. Results refer to $T = 20$.

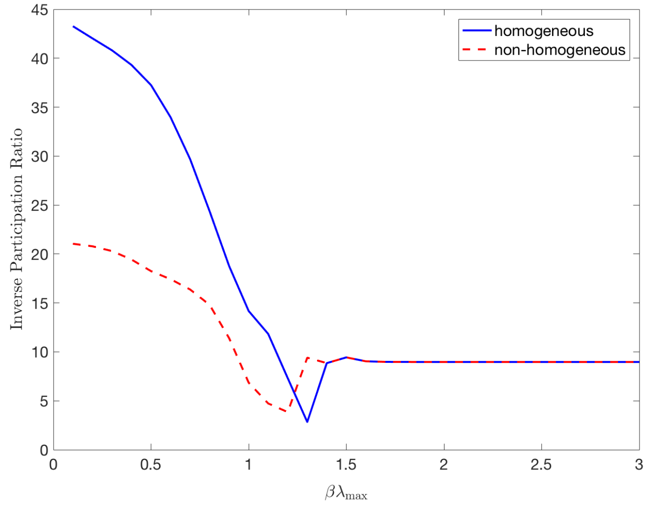

Supplementary Figure 13: Inverse participation ratio as a function of $\beta\lambda_{\max}$: comparison between the case where $\ell_i = 0.5$ for all nodes vs. the case where ℓ_i is drawn from a uniform distribution between zero and one. Results refer to $T = 20$.

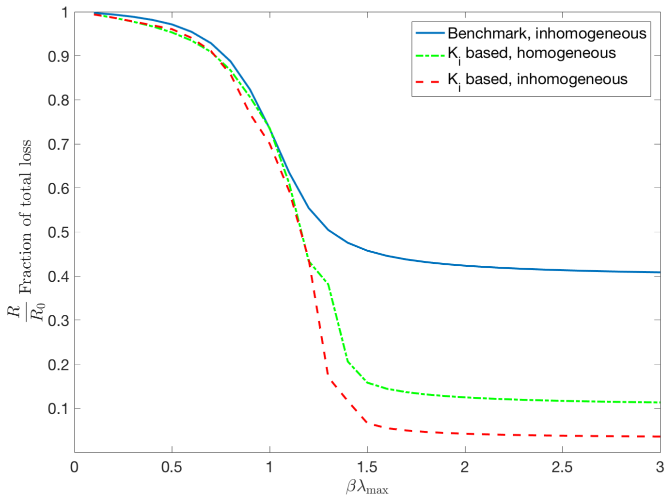

Supplementary Figure 14: Effect of policy intervention: comparison between the case where $\ell_i = 0.5$ for all nodes vs. the case where ℓ_i is drawn from a uniform distribution between zero and one. Results refer to $T = 20$.

References

[1] K. Anand, I. van Lelyveld, Á. Banai, S. Friedrich, R. Garratt, G. Hałaj, J. Fique, I. Hansen, S. M. Jaramillo, H. Lee, et al. The missing links: A global study on uncovering financial network structures from partial data. *Journal of Financial Stability*, 2017.

[2] M. Bacharach. Estimating nonnegative matrices from marginal data. *International Economic Review*, 6(3):294–310, 1965.

[3] S. Battiston, G. Caldarelli, M. D'Errico, and S. Gurciullo. Leveraging the network: a stress-test framework based on debtrank. *Statistics & Risk Modeling*, 33(3-4):117–138, 2016.

[4] N. Musmeci, S. Battiston, G. Caldarelli, M. Puliga, and A. Gabrielli. Bootstrapping topological properties and systemic risk of complex networks using the fitness model. *Journal of Statistical Physics*, 151(3):720–734, May 2013.
